# Detecting Visual Function Abnormality with a Contrast-Dependent Visual Test in Patients with Type 2 Diabetes

**DOI:** 10.1371/journal.pone.0162383

**Published:** 2016-09-09

**Authors:** Li-Ting Tsai, Kuo-Meng Liao, Yuh Jang, Fu-Chang Hu, Wei-Chi Wu

**Affiliations:** 1 Department of Occupational Therapy, Graduate Certificate in Low Vision Rehabilitation, University of Alabama, Birmingham, United States of America; 2 School of Occupational Therapy, College of Medicine, National Taiwan University, Taipei, Taiwan; 3 Department of Endocrine and Metabolism, Zhong-Xiao Branch, Taipei City Hospital, Taipei, Taiwan; 4 Graduate Institute of Clinical Medicine, College of Medicine, National Taiwan University, Taipei, Taiwan; 5 Department of Ophthalmology, Chang Gung Memorial Hospital and Chang Gung University, School of Medicine, Taoyuan County, Taiwan; Save Sight Institute, AUSTRALIA

## Abstract

In addition to diabetic retinopathy, diabetes also causes early retinal neurodegeneration and other eye problems, which cause various types of visual deficits. This study used a computer-based visual test (Macular Multi-Function Assessment (MMFA)) to assess contrast-dependent macular visual function in patients with type 2 diabetes to collect more visual information than possible with only the visual acuity test. Because the MMFA is a newly developed test, this study first compared the agreement and discriminative ability of the MMFA and the Early Treatment Diabetic Retinopathy Study (ETDRS) contrast acuity charts. Then symbol discrimination performances of diabetic patients and controls were evaluated at 4 contrast levels using the MMFA. Seventy-seven patients and 45 controls participated. The agreement between MMFA and ETDRS scores was examined by fitting three-level linear mixed-effect models to estimate the intraclass correlation coefficients (ICCs). The estimated areas under the receiver operating characteristic (ROC) curve were used to compare the discriminative ability of diseased versus non-diseased participants between the two tests. The MMFA scores of patients and controls were compared with multiple linear regression analysis after adjusting the effects of age, sex, hypertension and cataract. Results showed that the scores of the MMFA and ETDRS tests displayed high levels of agreement and acceptable and similar discriminative ability. The MMFA performance was correlated with the severity of diabetic retinopathy. Most of the MMFA scores differed significantly between the diabetic patients and controls. In the low contrast condition, the MMFA scores were significantly lower for 006Eon-DR patients than for controls. The potential utility of the MMFA as an easy screening tool for contrast-dependent visual function and for detecting early functional visual change in patients with type 2 diabetes is discussed.

## Introduction

The worldwide increase in the prevalence of diabetes mellitus (DM) has become an important public concern in both developed and developing countries [1]. Diabetic retinopathy (DR) is one of the most common complications of DM and the leading cause of visual impairment and blindness in the working-age population [2]. Although DR is characterized as a microvascular disease [3], recent studies have suggested that in the early stage of disease progression, diabetes causes retinal neurodegeneration [4, 5] and other different types of visual functional deficits and visual problems [4, 6, 7].

Retinal neurodegeneration, such as alterations in the retinal ganglion cells and inner retinal neurons, can cause various forms of visual deficits, such as decreased contrast sensitivity and altered color and temporal perception. These deficits may occur before changes in vascular morphology and visual acuity (VA) [4, 5, 8]. The current DR screening tools, including ophthalmoscopy, digital fundus photography, optical coherence tomography, and fluorescent angiography, principally assess the morphologic integrity of retina and retinal circulation [9, 10]. In addition, the VA is assessed with high-contrast optotypes. Therefore, these tools may be unable to detect early visual functional changes under lower contrast conditions in diabetic patients. Although these early visual functional changes are possibly due to retinal neurodegeneration, they also possibly come from other eye problems caused by diabetes, such as tear-film changes [6, 7], medial opacity [11], retinal neuro-sensory disturbance, or optic nerve dysfunction [4, 5]. Thus, if only VA testing and current DR screening tools are used, early visual disturbances in diabetic patients may not be detected.

In addition to VA, another important parameter in spatial vision is contrast sensitivity, which has been extensively investigated in diabetic patients [12–16]. Several studies have shown early abnormalities in low contrast discrimination or contrast sensitivity in diabetic patients [12, 15, 16]. The retinal sensitivity, assessed by microperimetry [17, 18], and the retinal temporal contrast threshold, assessed by flicker perimetry [19], have demonstrated changes in the visual function of DM patients not only in the fovea but also in the region outside it. Furthermore, the reduction of temporal vision in patients with diabetes has been noted in several studies [20, 21]. Temporal vision refers to how the time course of the stimuli affects the observer’s visual perception, including whether the features of visual stimuli can be integrated and how visual stimuli are perceived during a limited time window [22]. These functional visual tests are usually performed separately, and the integration of these functions into a test has not been reported to date.

To address these issues of visual dysfunction in diabetic patients, we designed a test for visual function, the Macular Multi-Function Assessment (MMFA), to measure the performance of contrast-dependent visual discrimination, assess the macular region instead of only the foveal area, and limit the presentation time of visual stimuli to include the factor of temporal integration or summation measurement during symbol recognition in the test. The MMFA is a computer program, the basic structure and content of which are based on the Macular Mapping Test (MMT) [23]. Although the MMFA may have several advantages in the assessment of visual function, owing to its initial application in diabetic patients, the first purpose of the current study was to examine the agreement between scores on the MMFA and ETDRS contrast acuity charts in diabetic patients and controls and to compare the abilities of the two tests to differentiate between the diabetic and control groups. The ETDRS contrast acuity charts, regarded as the criterion in contrast-dependent visual testing in this study, are frequently used to measure foveal contrast acuity in research and clinical applications [12, 15, 24]. Although the ETDRS charts test only the foveal function of the patients, not the whole macula, the good agreement and similar discriminative ability between the MMFA and ETDRS charts would help to validate the MMFA. The second purpose of the study was to examine whether macular visual function as measured by the MMFA in different contrast conditions would differ significantly between diabetic patients with different stages of DR and controls.

## Methods

### Participants

All of the procedures adhered to the tenets of the Declaration of Helsinki and were reviewed and approved by the local Institutional Review Board of Taipei City Hospital. Informed consent was obtained from each participant after the procedures of this study had been thoroughly explained. All participants provided their written informed consent to participate in this study.

Diabetic participants were prospectively recruited from patients who regularly received treatment at the Department of Endocrine and Metabolism, Zhong-Xiao Branch, Taipei City Hospital, and who had registered for the Diabetes Share Care Network. Controls were selected from outpatients without diabetes mellitus, residents living near the hospital, hospital staff, and relatives and friends of the patients. Hospital record reviews, the past medical history, and the data from the regular health screening review were used to confirm that the controls did not have diabetes.

The Diabetes Share Care Network was established in 2001 in Taiwan to increase the quality of diabetes care and to reduce complications due to the disease. Hospitals enrolled in this program must be certified. Diabetic patients who participate in this network receive regular biochemical check-ups quarterly and ophthalmologic examinations from every month to once a year, depending on the condition of the eyes. Recruited diabetic patients were divided into five groups according to their retinopathy status: non-DR, NPDR, PDR, NPDR with CSME, and PDR with CSME. DR was graded according to the International Clinical Disease Severity Scale for DR. CSME was defined upon slit lamp biomicroscopy as “(1) thickening of the retina at or within 500 μm of the center of the macula; or (2) hard exudate at or within 500 μm of the center of the macula associated with thickening of adjacent retina; or (3) a zone of retinal thickening 1 disc area or larger, any part of which is within 1 disc diameter of the center of the macula”. The criteria were introduced by ETDRS and used in the current study [25, 26]. Two ophthalmologists were responsible for examining and categorizing the patients. These 2 ophthalmologists were attending staff with over 10 years of experience caring for diabetic patients.

The inclusion criteria for the diabetic patients included the following: (1) type 2 diabetes, (2) age between 20 and 80 years old, (3) membership in the Diabetes Share Care Network, and (4) regular ophthalmological and biochemical examinations. The exclusion criteria included: (1) presence of congenital colour vision defects, (2) spherical power worse than -6 diopters or cylinder power worse than -4 diopters [27], (3) clinical history or evidence of ocular or neurological diseases not caused by diabetes, including glaucoma, trauma, multiple sclerosis, stroke, Parkinson’s disease and Alzheimer’s disease, (4) treatment with medications which would influence visual functioning, such as ethambutol, amiodarone, plaquenil, corticosteroids, and vigabatrin, and (5) inability to recognize Chinese characters.

The inclusion criteria for the controls included: (1) fundus and visual acuity examinations within the past 6 months and (2) age between 20 and 80 years old. The exclusion criteria included: (1) presence of congenital colour vision defects, (2) refractive errors larger than six spherical and four cylinder dioptres, (3) clinical history or evidence of ocular diseases or conditions, including glaucoma, laser eye surgery, age-related macular degeneration, retinitis pigmentosa, and trauma, (4) clinical history of neurological diseases having the potential to affect contrast sensitivity, including multiple sclerosis, stroke, Parkinson’s disease, and Alzheimer’s disease, (5) treatment with medications which would influence visual functioning, such as ethambutol, amiodarone, plaquenil, corticosteroids, and vigabatrin, (6) inability to recognize Chinese characters, and (7) a lack of diabetes.

### Macular Multi-Function Assessment (MMFA)

The MMFA is a computer program that allows variation of the settings of the contrast level based on the Weber contrast formula, the size and type of the optotypes, the presentation duration of the optotypes, and the size of the central fixation point. The basic structure of the MMFA is based on that of the MMT [23], which uses an eight-spoke wagon wheel to help stabilize the observer’s fixation during assessment. However, the MMFA provides new functions, including the ability to upload any optotypes (e.g., alphabetical letters, Chinese characters, Japanese characters, numbers, or symbols such as Lea symbols or animal symbols), an adjustable target size with consideration of the factors of eccentricity and meridians, and gamma correction in contrast measurement [28]. The targets used in the MMT are limited to the Landolt C, Snellen E, or letters. Although the Landolt C and Snellen E are standardized symbols used for testing vision, they may, under certain conditions, be unsuitable for representing the visual capacity for non-alphabetic languages such as Chineses [29]. The meridian factor is considered in the MMFA but not addressed in the MMT. Nonuniform visual spatial sampling, such as the effects of horizontal-vertical anisotropy and vertical meridian asymmetry, was observed in a prior study [29]. The function of luminance and voltage of an electronic display follows not a linear but a gamma curve, so it needs to be linearised with gamma correction to ensure correct luminance output [28]. Therefore the gamma correction provided by the MMFA allows a higher level of precision in luminance reproduction.

The radius of the MMFA is 12° of visual angle, and the testing platform has 41 testing locations. A single optotype is randomly presented in the fovea (0° and 1°) and the peripheral positions of 2°, 4°, 6°, and 8° along 8 axes (8 axes × 5 positions (1°, 2°, 4°, 6°, and 8°) plus 0° twice, for a total of 42 measuring points). For scoring, correct identification is given 2 points, incorrect identification (naming incorrectly) is given 1 point, detecting only the presence of a blurred image is given 0.5 points, and no detection at all is given 0 points. For example, if the participant identifies the character 仕 as 仟 or 江, 1 point (incorrect identification) is given. If the participant reports only detecting a blurred image and cannot clearly identify its pattern, 0.5 points are given. Thus, the total possible score of the MMFA is 84. Before the MMFA is used, the screen width and height are set to calculate the viewing distance. S1 Fig demonstrates the MMFA testing plate (S1A Fig) and the result of macular function assessment in JPEG format in a healthy participant (S1B Fig) as well as a type 2 diabetic patient with proliferative diabetic retinopathy tested at 25% (S1C Fig) and 10% (S1D Fig) contrast. In S1B to S1C Fig, white dots represent correct identification (2 points), grey dots represent incorrect responses—including incorrect identification (1 point) or detecting a blurred image (0.5 points), and black dots indicate that the participant did not detect anything at all (0 points).

#### Visual stimuli

In this study, traditional Chinese characters were used as optotypes to measure macular function. The main reason for using Chinese characters instead of using E, C, Sloan letters, or numbers, was that Chinese characters may have been more suitable to represent the visual capacity of our participants, whose primary language was Chinese. The stimuli in this study consisted of five sets of traditional Chinese characters. Each set contained 2 to 3 characters, and the characters in each set had similar configurations, stroke densities, and stroke frequencies. The stroke density was expressed as a pixel ratio of bit maps of the character images: the total pixels of the strokes to whole background image pixels. The stroke frequency was determined by a horizontal slice through each character at half the ascender height, and counting the number of lines crossed by the slice (strokes/character) [30]. The average number of stroke lines across all characters was 2.21. The stroke frequency in terms of strokes per character was also converted to strokes per degree as the stroke number divided by the letter width. Therefore, the mean stroke frequencies (stroke/degree) of stimuli at the fovea were 6.31, 4.60, 3.16, and 2.83 for the 80%, 25%, 10%, and 5% contrast levels respectively. The fourteen characters used in this study and their configurations and stroke densities are shown in S1 Table.

The size of the optotypes presented on each location (total of 41 locations) of the MMFA testing plate was not constant and was determined by the testing contrast level, its meridian dimension, and its eccentricity position. The reason is that these factors affect the size threshold required to discriminate Chinese characters. For example, because central VA is better than peripheral VA, a character at the periphery will be larger than a character at the foveal region. The relationship between the character size and eccentricity position is described as:

$$\omega_{T}=\;\omega_{T_{0}}\;\times \;\left(1+\;\frac{E}{E_{2}}\right)$$

where ω_*T*_ is the character size threshold at the eccentricity *E*, ω_*T0*_ is the size threshold in the centre of the fovea, and *E*_*2*_ represents the cortical magnification factor [31]. Different contrast levels and meridian dimensions had different ω_*T0*_ and *E*_*2*_ values. The values of ω_*T0*_ and *E*_*2*_ were determined in our previous psychophysical study of healthy participants with normal or corrected-to-normal VA, and the values used in this study are shown in S2 Table [32]. Therefore, the character sizes of the visual stimuli at the center of the fovea were 0.35, 0.48, 0.70, and 0.78 degrees of visual angle for the 80%, 25%, 10%, and 5% contrast levels respectively.

#### Apparatus of the MMFA

Stimuli were displayed on a ViewSonic monitor (G90fB 19”) driven by an ASUS computer with an Intel Core i7 display card. Luminance was measured with a Minolta LS-110 luminance meter, and gamma correction was performed with the MMFA software. The luminances of the black and white background were 0.53 cd/m^2^ and 127.9 cd/m^2^ respectively.

#### Protocol of the MMFA

The protocol of the MMFA in this study used four runs corresponding to four contrast levels (80%, 25%, 10%, and 5% contrast). All testing started from high contrast condition (80%), and other three testing conditions were then randomized. The viewing distance was 103.5 cm. All the testing was performed on the eye with better VA or, when both eyes had equal VA, on the preferred eye as determined by the Miles test [33].

Participants wore their up-to-date corrected eyeglasses at the time of the study. They had their chins supported when they performed the test. None of the patients wore bifocal or multi-focal lenses. They were instructed to maintain fixation on the central fixation point of the wagon wheel (cross-shaped, size: 0.2 degree of visual angle, luminance: 38.8 cd/m^2^) or, for patients who could not detect the central fixation point, on the centre area of the wagon wheel. Detailed instructions were given to the patients before the test was carried out to ensure that the patients understood the scoring criteria completely. Patients were encouraged to try their best to identify the target even if the target appeared to be foggy. In addition, before taking the actual test, the patients completed one practice session of the test to demonstrate their understanding of the scoring criteria. Starting from the test, participants told the examiner which character they perceived, and the examiner pressed a key corresponding to the verbal response of the participant. This key press also triggered the beginning of the next trial. The central fixation point was on constantly to help examiners maintained their fixation and off only when the character was presented at the foveal location. Each character was presented for 250 ms, which was long enough for the observer to discriminate the character and reduce the influence of saccades that would affect the assessment [23, 34, 35]. The interval between each key press and presentation of the next character was 500 ms. Each run lasted for 5 to 6 minutes.

### ETDRS contrast acuity charts

Contrast letter acuity (100%, 25%, 10%, and 5% contrast levels) was measured monocularly and binocularly in a standardized procedure at 3 m using the ETDRS PV Numbers Translucent Vision Charts from Precision Vision. Only the contrast acuities of the better eyes are presented in this study. All testing started from high contrast acuity, and other acuity tests were then randomized. Each line on the charts had five optotypes, and testing was stopped when participants made three or more errors on one line. The scoring was based on the total number of optotypes read correctly. All tests were performed at a constant background luminance of 85–90 cd/m^2^.

### Statistics analyses

The statistical analysis was performed using the R 3.1.2 software (R Foundation for Statistical Computing, Vienna, Austria), SPSS 13.0 for Windows (SPSS Inc., Chicago, IL, USA), and SAS 9.2. In the statistical testing, a two-sided *p* value ≤ 0.05 was considered statistically significant. The distributional properties of continuous variables were expressed by mean ± standard deviation (SD), whereas categorical variables were presented as frequencies and percentages.

To examine if the MMFA test could work as well as the ETDRS test, we performed two statistical analyses. First, we fitted a four 2-level mixed effects model to estimate the contrast-specific intraclass correlation coefficients (ICCs) and their 95% confidence intervals (CIs) respectively, followed by a one 3-level linear mixed-effects model to estimate the pooled ICCs across four contrast levels for examining the agreement between those two tests [36]. An ICC ≥.70 indicates an acceptable level of agreement between the two tests for comparison. Second, we fitted two multiple logistic regression models of DM versus non-DM, DR versus non-DR, and CSME versus non-CSME participants, respectively, to estimate the areas under the receiver operating characteristic (ROC) curve (also called the *c* statistic) and their 95% CIs to compare the ability to discriminate diseased and non-diseased patients between those two tests. The covariates considered in the logistic regression analyses were age, cataract status, contrast conditions, hypertension, HbA1c, duration of diabetes, and some of their interactions. A value of the estimated area under the ROC curve (AUC) ≥ 0.7 indicates an acceptable level of discrimination power.

For comparisons of baseline and clinical characteristics among the groups at different stages of DR and between the diabetic patients and controls, independent *t*-tests or one-way ANOVA were applied if the variables were continuous, and the Chi-square test was used if the variables were categorical. Pearson’s correlation was also used to measure the strength of agreement between the normalized scores of the MMFA and ETDRS contrast acuity charts at high contrast (80% for MMFA, 100% for ETDRS), and at the 25%, 10%, and 5% contrast levels. An absolute value of the correlation coefficient ranging from 0.50 to 0.75 indicated moderate to good correlation, and values above 0.75 were considered to indicate good to excellent correlation [37].

Multiple linear regression analysis was used to compare the MMFA scores (at the 80%, 25%, 10%, and 5% contrast levels) between the DR groups and non-DR patients, after adjusting for the effects of potential confounding factors, including age, sex, hypertension, duration, biochemical data (HbA1c, T-CHO, LDL, HDL, GPT, creatinine, and triglyceride) and cataract status. Trend tests were also examined to test whether the performance of the MMFA worsened as the DR stage progressed, after adjusting for the effects of potential confounding factors such as age, sex, hypertension, duration, biochemical data and cataract status. To compare the MMFA scores (at 80%, 25%, 10%, and 5% contrasts) between the DM patients and controls, multiple linear regression analysis was used, after adjusting for the effects of potential confounding factors (age, sex, hypertension, and cataract status) and stratifying by age and cataract status for accurate analysis. The coefficient of determination, *R*^2^, was computed to assess the goodness of fit of a fitted linear regression model.

## Results

### Participant characteristics

A total of 84 diabetic patients and 49 non-diabetic controls met the inclusion criteria and were enrolled in this study. However, only 77 patients and 45 controls finished all the visual tests. Four diabetic patients were excluded due to congenital colour vision impairment (*n* = 4), and two, due to the inability to recognize Chinese characters (*n* = 2) and thus finishing only the ETDRS test. One patient withdrew from assessment due to psychological problems after finishing only part of the ETDRS test. Three controls were excluded due to high-degree myopia (*n* = 1), glaucoma (*n* = 1), and a lack of data of recent ophthalmological examinations (*n* = 1). One control finished only the ETDRS test due to an inability to recognize Chinese characters (*n* = 1).

Table 1 presents all the participants’ baseline characteristics. Table 2 shows the baseline characteristics and biochemical data of the diabetic patients, including duration of diabetes, baseline biochemical data when they were recruited into the Diabetes Share Care Network, and eye health status at the most recent ophthalmological examination. Because the controls did not have diabetes mellitus, regular blood biochemical data could not be obtained. Significant differences were observed in age and in the status of hypertension and cataracts between the diabetic patients and controls. The criteria of hypertension were defined as blood pressure greater than 140/90 mmHg or treatment with anti-hypertensive drugs. Of the 77 diabetic patients, 64% had hypertension and 66% had cataracts. Of the controls, 29% had hypertension and 18% had cataracts. Age, sex, and cataract and hypertension status were likely to affect the performance of visual function, so they were adjusted for in the multiple linear regression analysis. Baseline and clinical characteristics were similar in the different DR groups except for disease duration. There were no significant differences in biochemical data among the different stages of the DR groups.

**Table 1:** Participant characteristics.

Variable	Type 2 Diabetes (*n* = 77)	Non-diabetic Control (*n* = 45)	*p* value
Age			
≤ 60 y/o	37 (48.1%)	26 (52.5%)	0.300
> 60 y/o	40 (51.9%)	19 (47.5%)	
Age (years)	59.5 ± 10.5 (22~78)	54.5 ± 15.4 (23~74)	0.037
Sex			
male	46 (59.7%)	24 (53.3%)	0.477
female	31 (40.3%)	21 (46.7%)	
Visual acuity	0.205 ± 0.270	-0.002 ± 0.124	<0.001
Hypertension			
yes	48 (63.6%)	13 (28.9%)	0.001
no	29 (36.4%)	32 (71.1%)	
Cataract			
normal	20 (26.0%)	35 (77.8%)	0.001
cataract	51 (66.2%)	8 (17.8%)	
s/p cataract surgery	6 (7.8%)	2 (4.4%)	

Continuous variables are presented as mean (SD); the difference between the diabetics and controls was tested using the independent *t*-test. Categorical variables are presented as *n* (%); the difference between diabetics and controls was tested using the Chi-squared test.

**Table 2:** Baseline characteristics and biochemical data of the diabetic patients by group.

Variables	Non-DR (*n* = 37)	NPDR (*n* = 22)	PDR (*n* = 4)	ME & NPDR (*n* = 8)	ME & PDR (*n* = 6)	*p* value
Age (years)	57 (12.3)	62 (8.5)	56 (10.7)	62 (5.6)	64 (7.4)	0.300
Sex						
male	20 (54.1)	12 (54.5)	4 (100.0)	7 (87.5)	3 (50.0)	0.182
female	17 (45.9)	10 (45.5)	0 (0.0)	1 (12.5)	3 (50.0)	
Visual Acuity	0.12 (0.17)	0.18 (0.23)	0.12 (0.09)	0.36 (0.22)	0.72 (0.42)	<0.001*
Disease duration (years)	6.6 (4.6)	12.4 (6.9)	14.8 (8.3)	15.8 (6.2)	14.0 (6.3)	<0.001*
Hypertension						
yes	20 (54.1)	13 (59.1)	4 (100.0)	7 (87.5)	5 (83.3)	0.142
no	17 (45.9)	9 (40.9)	0 (0.0)	1 (12.5)	1 (16.7)	
HbA1c (%)	8.5 (2.0)	9.2 (2.4)	10.0 (2.9)	8.2 (1.0)	8.5 (3.4)	0.539
HbA1c (mmol/mol)	70	77	85	66	70	
T-CHO	190 (45.4)	210 (82.6)	198 (40.1)	180 (33.9)	207 (38.9)	0.656
Triglyceride	155 (72)	316 (657)	274 (260)	153 (80)	153 (64)	0.525
LDL	114 (36.8)	109 (40.6)	99 (14.7)	102 (34.5)	121 (28.4)	0.786
HDL	45 (11.5)	46 (11.4)	50 (22.3)	44 (10.3)	56 (10.4)	0.309
GPT	38 (30.2)	36 (40.9)	21 (10.0)	41 (38.2)	19 (7.4)	0.660
Creatinine	1.1 (1.4)	0.8 (0.2)	1.3 (0.5)	1.3 (0.5)	2.7 (0.8)	0.083
Cataract						
normal	14 (37.8)	3 (13.6)	1(25.0)	1 (12.5)	1 (16.7)	0.179
cataract	21 (56.8)	17 (77.3)	3 (75.0)	7 (87.5)	3 (50.0)	
s/p cataract surgery	2 (5.4)	2 (9.1)	0 (0.0)	0 (0.0)	2 (33.3)	

Continuous variables are presented as mean (SD); the difference between the diabetics and controls was tested using the independent *t*-test. Categorical variables are presented as *n* (%); the difference between diabetics and controls was tested using the Chi-squared test. The biochemical data included baseline data collected when the diabetic patients were first recruited into the Diabetes Share Care Network. HbA1c: Hemoglobin A1c; T-CHO: Total cholesterol; GPT: Glutamic-Pyruvic Transaminase.

* indicates significant *p* values (*p* < 0.05).

### Agreement and discriminative ability between the scores of the MMFA and ETDRS charts

**Fig 1:**
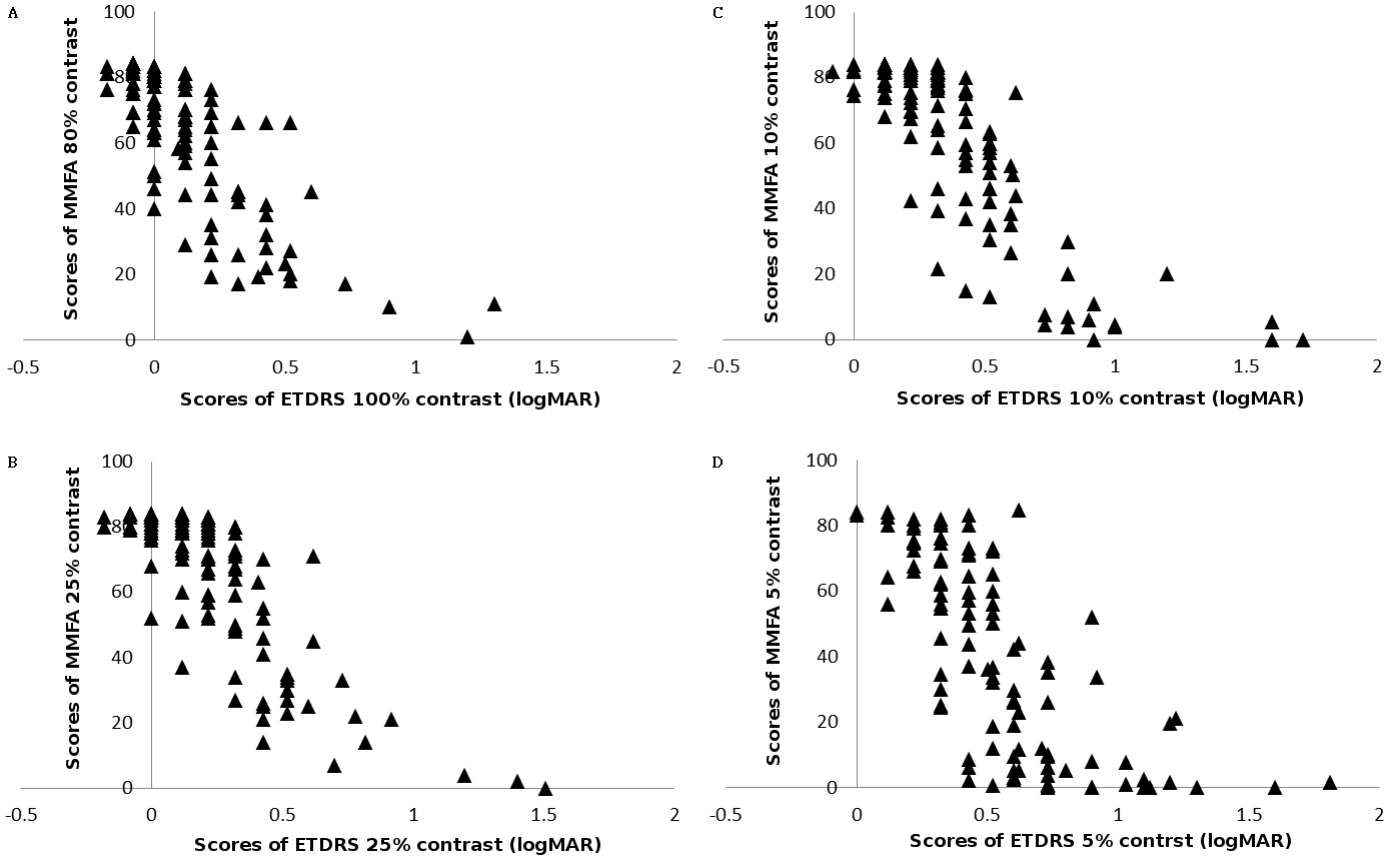
Scatter graphs demonstrating the correlations between the MMFA and EDTRS contrast acuity charts. High to moderate correlations shown at (a) high (100% for EDTRS charts, 80% for MMFA), (b) 25%, (c) 10%, and (d) 5% contrast levels.

Fig 1 illustrates the scatterplots of the scores of ETDRS contrast acuity charts with the MMFA at high (100% for ETDRS chart, 80% for MMFA), 25%, 10%, and 5% contrast levels for all participants. The scores of the ETDRS charts were transformed into a log of the minimum angle of resolution (LogMAR) scale.

To examine the agreement between the two tests, we fitted four 2-level mixed effects models (5 groups of subjects and 2 tests) to estimate the contrast-specific ICCs and 95% CIs respectively, and then fitted one 3-level linear mixed effects model (5 groups of subjects, 4 contrast levels, and 2 tests) to estimate the pooled ICCs across four contrast levels [36]. The contrast-specific ICCs were high at the contrast levels of 80%, 25%, and 10% (ICCs = 0.79, 0.82 and 0.82, respectively), but slightly lower at the contrast level of 5% (ICCs = 0.71). Nevertheless, the value of the pooled ICCs across four contrast levels was still high (ICC_pooled_ = 0.83). The computation process and program for estimating the pooled ICCs are shown in S1 Appendix. Clearly, some heterogeneity existed in the values of ICCs between the four contrast conditions.

Good to moderate correlation was found between 80% contrast of the MMFA and 100% contrast of the ETDRS chart (*r* = -0.79, *p* < 0.001), and the 25%, 10%, and 5% contrasts of the MMFA and the ETDRS chart (*r* = -0.82, *p* < 0.001; *r* = -0.82, *p* < 0.001; and *r* = -0.71, *p* < 0.001, respectively). The values of agreement (ICCs) and correlations (Pearson’s *r*) are shown in Table 3.

**Table 3:** Agreement and correlation of the MMFA and ETDRS tests at four contrast levels.

Test	ICCs (95% C.I.)	*r* (95% C.I.)
100% ETDRS vs. 80% MMFA	0.79 (0.71–0.85)	-0.79 (-0.71~-0.85)
25% ETDRS vs. 25% MMFA	0.82 (0.75–0.87)	-0.82 (-0.75~-0.87)
10% ETDRS vs. 10% MMFA	0.82 (0.75–0.87)	-0.82 (-0.75~-0.87)
5% ETDRS vs. 5% MMFA	0.71 (0.60–0.79)	-0.71 (-0.61~-0.79)

The results of the ROC curve analysis to differentiate participants with DM versus non-DM participants, patients with DR versus those without DR, and patients with CSME versus those without CSME are presented in Table 4. When all of the contrast conditions were incorporated into multiple logistic regression models, the MMFA test showed higher AUC values than those of the ETDRS in discriminating DM versus non-DM (0.827 versus 0.801) participants, and CSME versus non-CSME (0.821 versus 0.787) participants numerically. In the ROC analysis, which incorporated all potential predicting factors (age, hypertension, and cataract status in DM versus non-DM, and age, hypertension, cataract, duration, and HbA1c in DR versus non-DR, and CSME versus non-CSME participants), age, hypertension, and HbA1c did not significantly increased discriminative power, but the duration of diabetes and cataract status significantly increased the discriminative power and AUC in the ROC analysis. In the full models, which incorporated all the potential predicting factors and their interactions, the MMFA showed higher AUC values than those of the ETDRS tests in discriminating DM versus non-DM (0.940 versus 0.906), DR versus non-DR (0.951 versus 0.942), and CSME versus non-CSME (0.962 versus 0.928) participants numerically. S2 Fig presents six examples of the results of the ROC curve analysis to differentiate DM versus non-DM participants (S2A and S2B Fig), participants with DR versus non-DR (S2C and S2D Fig), and participants with CSME versus non-CSME (S2E and S2F Fig) in the full models in the MMFA and the ETDRS test.

**Table 4:** AUC (95% C.I.) of the ROC curve analysis to discriminate patients and controls, patients with DR and participants without DR, and patients with CSME and participants without CSME.

*X* (covariate)	*Y* (response variable)					
	DM vs. non-DM		DR vs. non-DR		CSME vs. non-CSME	
	MMFA	ETDRS	MMFA	ETDRS	MMFA	ETDRS
80% (100%)	0.780	0.816	0.869	0.903	0.808	0.779
25%	0.677	0.769	0.822	0.876	0.773	0.769
10%	0.719	0.797	0.884	0.860	0.792	0.784
5%	0.743	0.779	0.836	0.874	0.750	0.787
80% (100%), 25%, 10%, 5%	0.827	0.801	0.901	0.903	0.821	0.787
80% (100%), 25%, 10%, 5%, age	0.823	0.801	0.897	0.905	0.820	0.788
80% (100%), 25%, 10%, 5%, hypertension	0.826	0.809	0.907	0.919	0.829	0.809
80% (100%), 25%, 10%, 5%, cataract	0.862	0.851	0.904	0.913	0.857	0.822
80% (100%), 25%, 10%, 5%, HbA1c	-	-	0.854	0.866	0.752	0.747
80% (100%), 25%, 10%, 5%, duration	-	-	0.937	0.934	0.948	0.918
80% (100%), 25%, 10%, 5%, age, hypertension, cataract	0.881	0.865	0.904	0.914	0.862	0.829
80% (100%), 25%, 10%, 5%, age, hypertension, cataract, interaction*	0.940	0.906	0.922	0.930	0.870	0.846
80% (100%), 25%, 10%, 5%, age, hypertension, cataract, duration	-	-	0.938	0.936	0.958	0.929
80% (100%), 25%, 10%, 5%, age, hypertension, cataract, duration, interaction*	-	-	0.951	0.942	0.962	0.928

* interaction of cataract and four contrast conditions

### Contrast and retinopathy influenced performance on the MMFA

**Fig 2:**
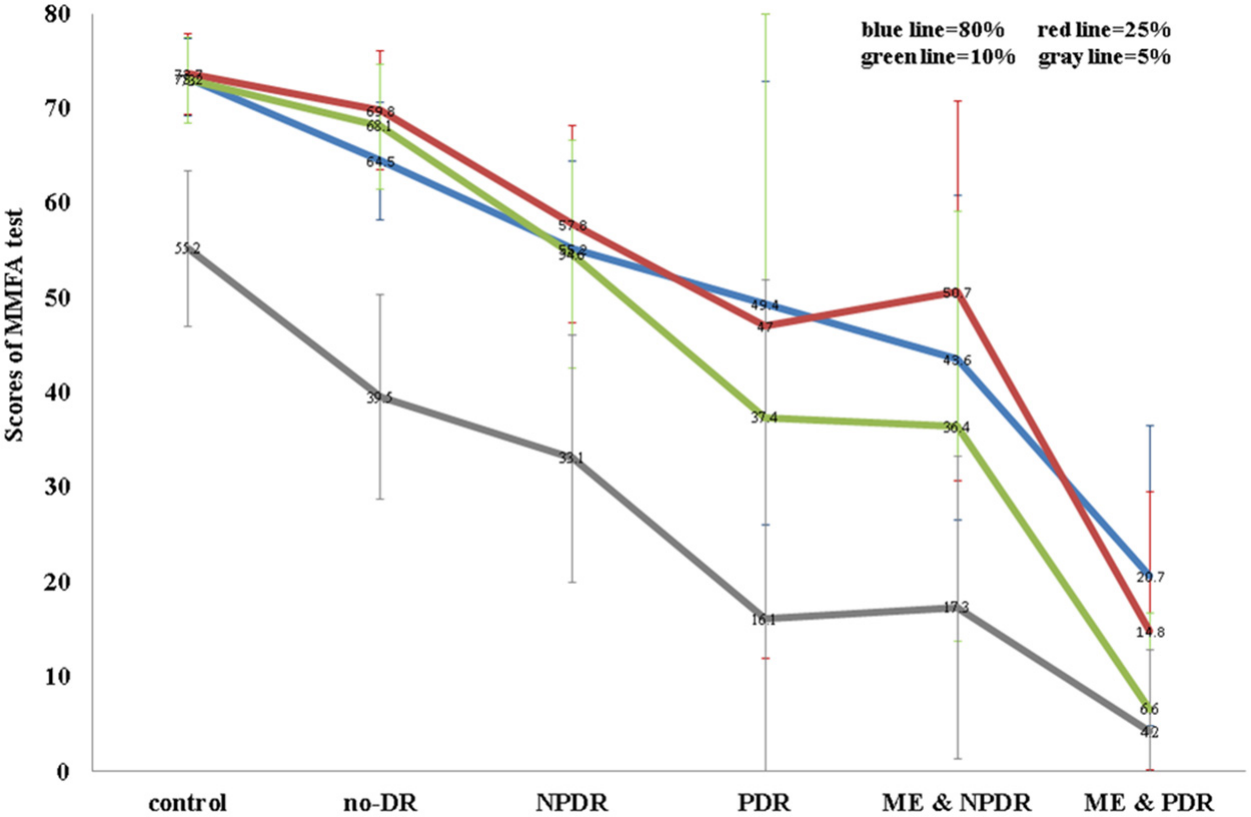
Mean scores of the MMFA in all participants by group. The total possible score of the MMFA is 84. (DR: diabetic retinopathy, NPDR: non-proliferative diabetic retinopathy, PDR: proliferative diabetic retinopathy, ME: macular edema).

The mean scores on the MMFA of all the participants are presented by group in Fig 2. The scores gradually decreased as the severity of DR increased. Multiple linear regression analysis was performed after adjusting for the effects of age, sex, duration of diabetes, biochemical data, cataract status, and hypertension status, to compare the MMFA scores among the diabetic patients (four DR groups versus the non-DR diabetic group). The non-DR group served as the reference group (Table 5). The MMFA scores of the PDR group were significantly lower than those of the non-DR group in the 10% contrast testing condition. The MMFA scores of the NPDR and CSME groups and the PDR and CSME groups were significantly lower than those of the non-DR group in all contrast testing conditions. There were no significant differences between the NPDR and non-DR groups at any contrast level. The *R*^2^ values were 0.53, 0.56, 0.55, and 0.53 for contrast levels of 80%, 25%, 10%, and 5% respectively. Trend tests were performed among the non-DR, NPDR, and PDR groups. After adjusting for the effects of potential confounding factors such as age, sex, hypertension, duration of diabetes, biochemical data, and cataract status, a significant trend was observed for all contrast conditions (*p* for trend *p* < 0.001). This means that as the severity of DR (non-DR, NPDR, and PDR) progressed, the MMFA scores decreased significantly.

**Table 5:** Multiple linear regression analysis of MMFA scores at 80%, 25%, 10%, and 5% contrast levels in different stages of DR groups.

Group	80% MMFA			25% MMFA			10% MMFA			5% MMFA		
	*β*	SE	*p* value	*β*	SE	*p* value	*β*	SE	*p* value	*β*	SE	*p* value
0												
1	-7.13	5.57	0.207	-9.25	6.03	0.130	-11.77	6.92	0.095	-10.57	8.14	0.200
2	-14.71	10.62	0.171	-21.38	11.50	0.068	-27.49	13.17	0.041*	-25.48	16.38	0.127
3	-19.95	7.92	0.015*	-17.79	8.58	0.043*	-28.73	9.82	0.005*	-24.20	11.43	0.040*
4	-42.89	8.81	<0.001*	-53.01	9.54	<0.001*	-53.14	12.03	<0.001*	-41.03	13.42	0.004*

Group 0 = no-DR; Group 1 = NPDR; Group 2 = PDR; Group 3 = NPDR & CSME; Group 4 = PDR & CSME. Data of the no-DR patients (Group 0) were used as the baseline for comparison with other diabetic groups (Groups 1 to 4).

* indicating significant p values (i.e., *p* < 0.05).

Furthermore, the MMFA scores of the DM groups and the control group were compared using multiple linear regression analysis after adjusting for age, sex, hypertension, and cataract status, and further stratified by age (≤ 60 y/o and >60 y/o) and cataract status (without cataracts, and with cataracts, and s/p cataract surgery) (Table 6). Significant differences in the MMFA scores were observed among the NPDR, PDR, NPDR with CSME, and PDR with CSME groups compared with controls at all contrast levels. However, significant differences in MMFA scores between the non-DR groups and the control group were observed only at the 5% contrast level. The MMFA was able to discriminate differences among the NPDR, PDR, NPDR with CSME, and PDR with CSME groups and controls at all contrast levels, but the differences between the non-DR groups and controls could be detected by the MMFA only at the 5% contrast level. The *R*^2^ values were 0.47, 0.46, 0.49, and 0.46 for contrast levels of 80%, 25%, 10%, and 5% respectively. While stratified by age group, significant linear trends were observed in all contrast conditions for age group of ≤60 y/o. However, the linear trends were less consistent in all contrast conditions for the age group of >60 y/o. Significant or borderline significant differences were observed among all the DM patient groups as compared with controls at all contrast levels in the age group of ≤60 y/o. For the age group of >60 y/o, a significant difference was observed only among the PDR with CSME group and the controls at the 80%, 25%, and 10% contrast levels. Stratified by cataract status, the linear trend became inconsistent in each subgroup, possibly due to the smaller sample size. Significant differences in the non-cataract group were mainly observed among the NPDR with CSME, PDR with CSME, and control groups. There were no significant differences observed between the NPDR, non-DR, and control groups. For the group with cataract or s/p cataract surgery, significant differences were observed among several DR groups and controls at the 80%, 25%, 10%, and 5% contrast levels.

**Table 6:** Multiple linear regression analysis of the MMFA scores at 80%, 25%, 10%, and 5% contrast levels for all participants.

Group	80% MMFA			25% MMFA			10% MMFA			5% MMFA		
	*β*	SE	*p* value	*β*	SE	*p* value	*β*	SE	*p* value	*β*	SE	*p* value
0												
1	-7.44	3.75	0.050	-1.73	4.04	0.670	-2.70	4.49	0.540	-12.67	5.73	0.029*
2	-14.33	4.60	0.002*	-10.96	4.96	0.029*	-13.10	5.38	0.017*	-14.67	6.68	0.030*
3	-21.89	8.64	0.013*	-23.62	9.32	0.013*	-32.90	10.12	<0.001*	-37.86	12.27	0.003*
4	-25.03	6.49	<0.001*	-17.26	7.00	0.015*	-30.58	7.60	<0.001*	-30.47	9.23	0.001*
5	-47.71	7.42	<0.001*	-52.36	8.00	<0.001*	-57.95	9.40	<0.001*	-41.19	10.61	<0.001*
Age ≤ 60 y/o												
0												
1	-13.67	4.37	0.003*	-8.64	4.99	0.089	-10.70	5.32	0.049*	-27.34	7.11	<0.001*
2	-17.19	6.21	0.008*	-14.90	7.09	0.040*	-19.32	7.56	0.013*	-37.15	9.60	<0.001*
3	-33.60	8.84	<0.001*	-35.52	10.09	<0.001*	-46.02	10.76	<0.001*	-58.72	13.25	<0.001*
4	-42.31	9.55	<0.001*	-38.70	10.90	<0.001*	-59.74	11.62	<0.001*	-75.58	14.46	<0.001*
5	-57.51	14.28	<0.001*	-58.22	16.29	<0.001*	-65.21	14.37	<0.001*	-79.84	21.34	<0.001*
Age > 60 y/o												
0												
1	-3.64	6.68	0.589	2.68	6.99	0.703	2.07	7.52	0.784	0.21	8.92	0.981
2	-16.97	7.70	0.032*	-13.20	8.07	0.108	-13.91	8.68	0.115	-1.01	10.00	0.920
3	-3.90	19.64	0.843	-7.72	20.57	0.709	-16.90	22.13	0.449	-22.27	25.30	0.383
4	-19.026	9.96	0.062	-8.70	10.43	0.408	-18.46	11.22	0.106	-6.18	12.82	0.632
5	-45.30	10.12	<0.001*	-50.54	10.60	<0.001*	-54.96	11.40	<0.001*	-25.31	13.10	0.059
No-cataract												
0												
1	-3.42	7.21	0.637	3.67	7.90	0.644	6.92	8.74	0.431	12.53	9.51	0.193
2	-14.58	7.51	0.057	-9.34	8.22	0.261	-7.67	9.11	0.403	5.64	9.67	0.562
3	-24.40	12.55	0.057	-27.64	13.74	0.049*	-35.84	15.21	0.022	-25.51	16.11	0.119
4	-23.46	9.43	0.017*	-12.80	10.33	0.220	-24.24	11.44	0.038*	-14.23	12.11	0.245
5	-50.17	10.12	<0.001*	-52.35	11.08	<0.001*	-49.35	13.05	<0.001*	-21.93	12.95	0.096
Cataract and s/p cataract surgery												
0												
1	-12.86	3.81	0.002*	-7.40	3.93	0.066	-10.87	3.72	0.005*	-30.01	7.08	<0.001*
2	-9.94	7.36	0.183	-9.15	7.60	0.234	-15.64	7.19	0.035*	-31.23	12.12	0.014*
3	-13.42	12.26	0.279	-3.88	12.65	0.760	-5.48	11.98	0.649	-19.85	20.15	0.330
4	-30.89	12.32	0.016*	-28.50	12.72	0.030*	-30.08	12.05	0.016*	-16.18	20.26	0.429
5	-26.65	12.48	0.038*	-38.78	12.88	0.004*	-64.72	12.20	<0.001*	-49.62	20.58	0.020*

Group 0 = non-DM control; Group 1 = no-DR; Group 2 = NPDR; Group 3 = PDR; Group 4 = NPDR & CSME; Group 5 = PDR & CSME. Data of the controls (Group 0) were used as the baseline for comparison with the diabetic patients (Groups 1 to 5) with different retinal conditions.

* indicating significant p values (i.e., *p* < 0.05).

## Discussion

To the best of our knowledge, the present study is the first to use a visual function test integrating multiple components (spatial and temporal vision) to investigate macular visual function in patients with type 2 diabetes. Significant trends in the MMFA scores were observed among the non-DR, NPDR, and PDR groups, and significant group differences among all diabetic groups and the control group were observed at the 5% contrast level. The non-DR group, despite having normal macular morphology, had lower scores on the MMFA in low contrast conditions. This difference may have been due to tear-film changes, medial opacity, retinal neuro-sensory disturbance, optic nerve dysfunction, or other potential non-visual factors, such as impaired cognitive ability [38, 39]. Although several potential factors may have affected the diabetic patients’ performance on the MMFA, this finding suggests that the contrast-dependent test of the MMFA may be a potentially useful screening tool for detecting early visual functioning changes in diabetic patients.

Many studies have shown that diabetes not only alters microvascular function, causing changes in vascular morphology, but also leads to media opacity and neurodegeneration, which in turn cause early visual problems such as impaired contrast and temporal perception [4, 5, 8, 40]. The diagnosis and classification of DR reflects only the severity of alterations in vascular morphology, and VA assessment measures only the visual response at high contrast levels. Thus, neither can comprehensively evaluate the potential visual problems caused by diabetes mellitus. Furthermore, visual dysfunction in diabetic patients is not limited to the foveal region, for it also affects other central areas [17, 19]. In comparison, the MMFA is able to assess high to low contrast-dependent visual performance, measure macular visual function instead of only that of the foveal region, and incorporate the temporal factor to improve the accuracy of visual function assessment for diabetic patients.

The superiority of the MMFA over previous tests for the assessment of contrast-dependent visual function in diabetic patients is supported by several findings of this study. When all of the contrast conditions were incorporated into the multiple logistic regression models, or all the potential predicting factors and interactions were incorporated into the full models, the MMFA showed higher AUC values than those of the ETDRS test in discriminating DM versus non-DM, DR versus non-DR, and CSME versus non-CSME participants numerically (Table 4). In addition, the results of multiple linear regression analysis of the ETDRS and MMFA scores at the 80%, 25%, 10%, and 5% contrast levels for all participants (S3 Table) showed that the MMFA can differentiate the controls and all DM groups at 5% contrast, and all DR groups at 80%, 25%, 10%, and 5% contrast. However, the results showed that the ETDRS test could significantly discriminate controls and the non-DR at 100% and 5% contrast, and controls and the NPDR groups at all contrast levels, but not the PDR group. A possible interpretation may be that although the PDR patients in this study had scattered spotty areas of visual loss, they still demonstrated good acuity when looking at a standard visual acuity chart with the best areas in their visual field, particularly in the unlimited testing time condition, which would influence the discriminative sensitivity of the ETDRS test. Similar result of the non-linear visual function changes along the severities of DR was observed in other study [12]. An example of a PDR patient with impaired function on the MMFA test who still performed well on the ETDRS test is provided in S3 Fig. Actually, while PDR patients perform well on a VA test, most have experienced difficulty with vision in their everyday life, particularly in lower contrast environments. Thus, a sensitive test to reflect a patient’s functional limitations will be very important for further clinical application.

Although the characteristics of the MMFA are different from those of the traditional chart tests, such as the ETDRS contrast acuity charts, its principle purpose is still to measure contrast vision. Agreement and correlation analysis with the ETDRS contrast acuity tests showed high agreement (ICC_pooled_ = 0.83) and significant correlation (*r* = -0.71 to -0.82) (Table 3). These values indicate that the MMFA has a high degree of similarity to the ETDRS charts in the measurement of contrast vision. However, the level of correlation did not exceed 0.9 [41], and therefore, we hypothesize that these outcomes may be related to the differences between these 2 tests. Although the MMFA and the ETDRS both determine the contrast acuity of the patients, the 2 tests have subtle differences. The MMFA measures not only the foveal region but also the entire macular area. In addition, there is a time constraint when using the MMFA, but no such limit with the ETDRS test. Therefore, the MMFA not only examines spatial factors, such as acuity (testing at high contrast level) and contrast sensitivity, but also involves the temporal factor, for the examinees have to respond within a set time limit. Prior studies have reported that the temporal factor is impaired in diabetic patients [20, 21], and the MMFA is a tool that could take this factor into consideration.

In the current study, significant trends were observed under all testing conditions in non-DR, NPDR, and PDR diabetic patients. That is, as the DR became more severe, the performance on the MMFA became worse. The trend was more predominant as the contrast level decreased. Performance on the MMFA can reflect the severity of DR.

When compared with the non-diabetes mellitus controls, the NPDR, PDR, NPDR with CSME, and PDR with CSME groups showed significantly lower scores in all contrast conditions. However, the non-DR group demonstrated a significant difference from the control group only at 5% contrast. The non-DR group, despite having normal macular morphology, had significantly lower scores on the MMFA in low contrast conditions. Similar findings have also been reported in other studies using another contrast test, the Pelli-Robson chart, to assess contrast sensitivity (CS) [12, 13]. Ismail and Whitaker demonstrated that the Pelli-Robson chart was able to distinguish the control group from each of the three DR groups (non-DR, early DR, and advanced DR) but unable to discriminate the differences among three diabetic groups [13]. In a study by Stavrou and Wood, the severity of DR was graded as level 1 (no DR or minimal DR), level 2 (mild DR), and level 3 (moderate or severe DR) [12]. A significant difference in the CS was found between the controls and the level 1 group, but not for the more severe groups of levels 2 and 3. In addition, neither study reported the characteristics of the participants, which may have affected the CS performance. In this study, we have shown that the MMFA can differentiate between the controls and all DM groups at 5% contrast, and between the controls and all the DR groups at the 80%, 25%, and 10% contrast levels. A significant trend was observed for all contrast levels. In addition, with detailed classification of DR, covariate adjustment, and stratified analysis, our study provides more reliable evidence than that of previous studies in support of the possibility of early visual function change in those with diabetes but without DR. However, diabetics and controls are different populations. Although we have adjusted for age, sex, hypertension, and cataract status for potential confounding factors, factors such as cognitive ability [38, 39], education level, blood glucose fluctuations [42], and diabetic neuropathy [43] were not included in the logistic models and may have influenced the tests results of the diabetic patients. These factors may also have the potential to influence testing performance on the MMFA. Taken together, the statistical difference between non-DR group and the non-DM group in low contrast conditions may be an indicator of early visual functioning change caused by diabetes mellitus, which may come from retinal neurodegeneration and other eye problems. However, this result may also have been due to other confounding factors not included in the statistical models, such as cognitive ability. Therefore, further studies including more potential confounding factors in the statistical analysis to validate the effects of low contrast testing in discriminating early visual change in non-DR diabetic patients will be necessary.

In our study, the proportions of participants with some characteristics (i.e., age and cataract status) differed between the groups with and without diabetes. Because those characteristics have been known to affect visual function and their effects could not be fully adjusted for in the multiple linear regression models, we performed stratified analysis by age group and cataract status to develop a more accurate analysis. In the younger age group (age ≤ 60 y/o), as shown in Table 6, all the DM groups had significantly lower scores in all contrast conditions (except the non-DR group, which at 25% contrast showed borderline significance) than did the non-DM controls. However, in the older age group (age> 60 y/o), only the scores of the PDR with CSME group were significantly lower than those of the controls. One possible explanation is that in the elderly group, many conditions may cause impaired contrast sensitivity (such as age-related macular degeneration, refractory error, neurodegeneration, and other eye conditions), which may alleviate the difference between the DM patients and controls [44–46]. However, in the younger age group, DM is the strongest factor, so the difference is more obvious. Stratification by cataract status revealed linear trends that were less consistent in all the contrast conditions due to the smaller sample size in each group. However, significantly lower MMFA scores were found in nearly all contrast conditions (5%, 10%, 80%) for the non-DR patients as compared to the controls in cataract group. No such phenomenon was found in the non-cataract group. As we know, cataracts may decrease contrast sensitivity, so they may interfere with the results of the MMFA and must be considered in future clinical applications of the MMFA.

The presentation time of the visual stimuli in the MMFA was limited to 250 ms to reduce the influence of saccadic eye movements [23]. This time duration has been shown to be sufficient for recognizing Chinese characters [35]. The rationale for limiting the amount of time for recognizing the characters was based on previous studies indicating abnormalities in the temporal perception of diabetic patients [20, 21]. In addition, in clinical studies and practice, the response time has also been used to monitor recovery and predict further outcomes [47], and visual environmental information is usually presented in a time-limited fashion. Therefore, including the temporal domain in the assessment can increase the accuracy of problem identification and open up further clinical applications.

The radius of the MMFA testing plate is 12° of the visual angle, and the MMFA has 41 testing locations equally distributed over the eight axes of the corresponding macular area. Although the main purpose of the MMFA is to measure the contrast dependence of visual function, another advantage of the MMFA is that it provides an inexpensive method of quickly and easily identifying and mapping local retinal abnormalities. There are several tests to represent parafoveal function, such as standard automated perimetry, microperimetry, and flicker perimetry, which use a light stimulus to detect scotoma in the macular area. In contrast, the MMFA uses character discrimination to test the patients’ contrast acuity. The other tests are also more complex, have a learning curve, and require the more cooperation of the patients [39]. The MMFA is simpler and easier to use at any assessment environment than these tests. Although the MMFA may not be as accurate as perimetric instruments, such as microperimetry or Humphrey perimeter program 10–2 for defining the areas of scotoma, a similar test, the Macular Mapping Test (MMT), has been reported to show high correlations with the results of manual perimetry [23]. Assessment of foveal contrast acuity alone may be inadequate to demonstrate visual dysfunction in diabetic patients because of parafoveal and perifoveal retinal impairment, even before the loss of VA and the onset of DR. In addition, local retinal impairment may be able to predict the development of new DR [17]. The MMFA may therefore be a potential tool for grossly screening lesion areas in clinical practice.

The main limitations of this study are as follows: (1) We did not collect the biochemical data of the control group, (2) the percentage of those with cataracts in the diabetic group was significantly higher than that in the control group, (3) sample sizes were small in the PDR, NPDR with CSME, and PDR with CSME groups, (4) the diopter difference when viewing the visual target at 1m and 3m was not considered, and (5) we did not record whether the patients had stable or unstable fixation during testing. The patients were instructed to maintain fixation on the central fixation point of the wagon wheel (cross-shaped, size: 0.2 degrees of visual angle, luminance: 38.8 cd/m^2^) or, for patients who could not detect the central fixation point, on the central area of the wagon wheel. The test was rehearsed before the official recording of the results. During the test, patients were also reminded of the importance of central fixation. However, we did not have an automatic tool to detect loss of fixation during the test, and this is a factor that could affect the study outcome. Since the control group underwent the same tests, the issue was corrected to a certain degree, if it did indeed affect the outcome. Further study will be required to determine the impact of fixation loss on the test.

Despite these limitations, no significant effect on the MMFA scores was detected among the various DR groups when the biochemical data were included into the multiple linear regression analysis. This suggests that the lack of biochemical data in the control group would not have significantly influenced the data analysis. In addition, although the sample sizes of the PDR, NPDR with CSME, and PDR with CSME groups were small, the results of the multiple linear regression analysis also indicated significant differences from the controls (see Table 6). Although the MMFA used the eight-spoke wagon wheel to help stabilize the observer’s fixation during assessment and a similar test, the MMT, has been reported to show high correlations with the results of manual perimetry [23], the current settings did not detect whether participants maintained stable fixation during testing. Therefore, further study combining eye tracking equipment and the MMFA for recording and computing online eye movement is needed.

Despite the various limitations of this study, the MMFA showed good correlation with the different stages of DR and the potential for detecting early visual functioning change in patients without DR. Undoubtedly, further studies with a larger number of diabetic patients with PDR, NPDR with CSME, and PDR with CSME, collecting more comprehensive data of confounding visual or non-visual factors related to influencing visual performance, and combining eye tracking with the MMFA for monitoring eye movement will be needed to confirm the properties of the MMFA and the interpretation of results in measuring macular visual function in diabetic patients.

## Supporting Information

S1 Appendix Details of the calculation of the pooled ICCs from the three-level linear mixed-effects model (DOCX)

S1 Fig The testing platform of the MMFA and testing results are shown in the figures. (A) Testing platform; (B) results of the MMFA in a representative healthy participant; (C) a type 2 diabetic patient with proliferative diabetic retinopathy tested at 25% contrast; and (D) the same patient tested at 10% contrast. The testing platform of the MMFA uses an eight-spoke wagon wheel to help stabilize the observer’s fixation. Visual stimuli are presented on the white wagon wheel (A). White dots represent correct identification (2 points). Grey dots represent incorrect responses (incorrect identification (1 point) or detection of a blurred image (0.5 point)). Black dots indicate that the participant did not detect anything at all (0 point) (B to D). In Fig 1(C), for example, there are 11 white dots (equal to 22 points), 20 grey dots (9 incorrect identifications (9 points) and 11 detecting blurred images (5.5 points)), and 11 black dots (0 point). Therefore, the total score of Fig 1(C) is 36.5 points. Compared with the control B) and between (C) and (D), this diabetic patient showed consistently poorer performance in the foveal, temporal, and lower fields. In addition, the more the contrast level decreased, the greater number of incorrect responses that occurred in the testing results.
(TIF)

S2 Fig Examples of the results of the ROC curve analysis to differentiate patients and controls in (A) the MMFA and (B) the ETDRS test, to differentiate participants with DR and without DR in (C) the MMFA and (D) the ETDRS test, and to differentiate participants with CSME and without CSME in (E) the MMFA and (F) the ETDRS test, in the full models. (TIF)

S3 Fig Examples of the results of a type 2 diabetic patient with proliferative diabetic retinopathy (PDR) tested using the MMFA at (A) 80% contrast (score = 46), (B) 25% contrast (score = 37), (C) 10% contrast (score = 21.5), and (D) 5% contrast (score = 6). The contrast acuity values tested by the ETDRS test at 100%, 25%, 10%, and 5% contrast were 1.00, 0.75, 0.32, and 0.43 (in decimal notation), respectively. Although this patient had PDR and impaired performance in the MMFA, he still performed well on the ETDRS test.
(TIF)

S1 Table Chinese character, configuration, and stroke density used in the MMFA. (PDF)

S2 Table Parameters (ω_*T0*_ and *E*_*2*_) used in the MMFA. (PDF)

S3 Table Comparing the results of multiple linear regression analysis of the ETDRS and MMFA scores at 80%, 25%, 10%, and 5% contrast levels for all participants. (PDF)

## References

[1] Zimmet P, Alberti KG, Shaw J. Global and societal implications of the diabetes epidemic. Nature. 2001; 414(6865):782–7. 10.1038/414782a . 11742409

[2] Ruta LM, Magliano DJ, Lemesurier R, Taylor HR, Zimmet PZ, Shaw JE. Prevalence of diabetic retinopathy in Type 2 diabetes in developing and developed countries. Diabet Med. 2013; 30(4):387–98. 10.1111/dme.12119 . 23331210

[3] Stitt AW, Lois N, Medina RJ, Adamson P, Curtis TM. Advances in our understanding of diabetic retinopathy. Clin Sci (Lond). 2013;125(1):1–17. 10.1042/cs20120588 . 23485060

[4] Antonetti DA, Barber AJ, Bronson SK, Freeman WM, Gardner TW, Jefferson LS, et al Diabetic retinopathy: seeing beyond glucose-induced microvascular disease. Diabetes. 2006; 55(9):2401–11. 10.2337/db05-1635 . 16936187

[5] Simo R, Hernandez C. Neurodegeneration is an early event in diabetic retinopathy: therapeutic implications. Br J Ophthalmol. 2012; 96(10):1285–90. 10.1136/bjophthalmol-2012-302005 . 22887976

[6] Bikbova G, Oshitari T, Tawada A, Yamamoto S. Corneal changes in diabetes mellitus. Curr Diabetes Rev. 2012;8(4):294–302. 22587515 10.2174/157339912800840479

[7] Kaiserman I, Kaiserman N, Nakar S, Vinker S. Dry eye in diabetic patients. Am J Ophthalmol. 2005; 139(3):498–503. 10.1016/j.ajo.2004.10.022 . 15767060

[8] Zhang X, Wang N, Barile GR, Bao S, Gillies M. Diabetic retinopathy: neuron protection as a therapeutic target. Int J Biochem Cell Biol. 2013; 45(7):1525–9. 10.1016/j.biocel.2013.03.002 . 23506699

[9] Hansen MB, Abramoff MD, Folk JC, Mathenge W, Bastawrous A, Peto T. Results of Automated Retinal Image Analysis for Detection of Diabetic Retinopathy from the Nakuru Study, Kenya. PLoS One. 2015;10(10):e0139148 10.1371/journal.pone.0139148 . 26425849 PMC4591009

[10] Cole ED, Novais EA, Louzada RN, Waheed NK. Contemporary Retinal Imaging Techniques in Diabetic Retinopathy: a review. Clin Experiment Ophthalmol. 2016 10.1111/ceo.12711 . 26841250

[11] Chen SJ, Liu JH, Shih HC, Chou P, Tsai CY, Tung TH. Prevalence and associated factors of lens opacities among Chinese type 2 diabetics in Kinmen, Taiwan. Acta Diabetol. 2008; 45(1):7–13. 10.1007/s00592-007-0012-9 . 17828461

[12] Stavrou EP, Wood JM. Letter contrast sensitivity changes in early diabetic retinopathy. Clin Exp Optom. 2003; 86(3):152–6. . 12767249 10.1111/j.1444-0938.2003.tb03097.x

[13] Ismall GM, Whitaker D. Early detection of changes in visual function in diabetes mellitus. Ophthalmic Physiol Opt. 1998; 18(1):3–12. 9666905

[14] Arend O, Remky A, Evans D, Stuber R, Harris A. Contrast sensitivity loss is coupled with capillary dropout in patients with diabetes. Invest Ophthalmol Vis Sci. 1997; 38(9):1819–24. 9286271

[15] Sukha AY, Rubin A. High, medium, and low contrast visual acuities in diabetic retinal disease. Optom Vis Sci. 2009;86(9):1086–95. . 19668018 10.1097/OPX.0b013e3181b48635

[16] Ewing FM, Deary IJ, Strachan MW, Frier BM. Seeing beyond retinopathy in diabetes: electrophysiological and psychophysical abnormalities and alterations in vision. Endocr Rev. 1998;19(4):462–76. 10.1210/edrv.19.4.0340 . 9715375

[17] Nittala MG, Gella L, Raman R, Sharma T. Measuring retinal sensitivity with the microperimeter in patients with diabetes. Retina. 2012; 32(7):1302–9. . 22450513 10.1097/IAE.0b013e3182365a24

[18] Verma A, Rani PK, Raman R, Pal SS, Laxmi G, Gupta M, et al Is neuronal dysfunction an early sign of diabetic retinopathy? Microperimetry and spectral domain optical coherence tomography (SD-OCT) study in individuals with diabetes, but no diabetic retinopathy. Eye (Lond). 2009; 23(9):1824–30. 10.1038/eye.2009.184 . 19648899

[19] Stavrou EP, Wood JM. Central visual field changes using flicker perimetry in type 2 diabetes mellitus. Acta Ophthalmol Scand. 2005; 83(5):574–80. 10.1111/j.1600-0420.2005.00527.x . 16187995

[20] Davies N, Morland A. Temporal visual filtering in diabetes mellitus. Vision Res. 2003; 43(22):2377–85. . 12962994 10.1016/s0042-6989(03)00405-x

[21] Gualtieri M, Bandeira M, Hamer RD, Damico FM, Moura AL, Ventura DF. Contrast sensitivity mediated by inferred magno- and parvocellular pathways in type 2 diabetics with and without nonproliferative retinopathy. Invest Ophthalmol Vis Sci. 2011; 52(2):1151–5. 10.1167/iovs.09-3705 . 21051718

[22] Hart WM Jr. The temporal responsiveness of vision In: MR A., HW M., editors. Adler's physiology of the eye: clinical application. 8 ed St. Louis, MO: Mosby; 1987 p. 429–57.

[23] Trauzettel-Klosinski S, Biermann P, Hahn G, Weismann M. Assessment of parafoveal function in maculopathy: a comparison between the Macular Mapping Test and kinetic manual perimetry. Graefes Arch Clin Exp Ophthalmo. 2003; 241(12):988–95. 10.1007/s00417-003-0757-y 14618339

[24] Balcer LJ, Galetta SL, Polman CH, Eggenberger E, Calabresi PA, Zhang A, et al Low-contrast acuity measures visual improvement in phase 3 trial of natalizumab in relapsing MS. J Neurol Sci. 2012; 318(1–2):119–24. 10.1016/j.jns.2012.03.009 . 22521274

[25] Wilkinson CP, Ferris FL 3rd, Klein RE, Lee PP, Agardh CD, Davis M, et al Proposed international clinical diabetic retinopathy and diabetic macular edema disease severity scales. Ophthalmology. 2003; 110(9):1677–82. 10.1016/s0161-6420(03)00475-5 . 13129861

[26] Grading diabetic retinopathy from stereoscopic color fundus photographs—an extension of the modified Airlie House classification. ETDRS report number 10. Early Treatment Diabetic Retinopathy Study Research Group. Ophthalmology. 1991; 98(5 Suppl):786–806. . 2062513

[27] Pitsas C, Papaconstantinou D, Georgalas I, Halkiadakis I. Relationship between short-wavelength automatic perimetry and Heidelberg retina tomograph parameters in eyes with ocular hypertension. Int J Ophthalmol. 2015;8(5):1013–7. 10.3980/j.issn.2222-3959.2015.05.29 26558219 PMC4631013

[28] To L, Woods RL, Goldstein RB, Peli E. Psychophysical contrast calibration. Vision Res. 2013; 90:15–24. 10.1016/j.visres.2013.04.011 . 23643843 PMC3744609

[29] Zhang JY, Zhang T, Xue F, Liu L, Yu C. Legibility variations of Chinese characters and implications for visual acuity measurement in Chinese reading population. Invest Ophthalmol Vis Sci. 2007; 48(5):2383–90. 10.1167/iovs.06-1195 . 17460306

[30] Majaj NJ, Pelli DG, Kurshan P, Palomares M. The role of spatial frequency channels in letter identification. Vision Res. 2002; 42(9):1165–84. . 11997055 10.1016/s0042-6989(02)00045-7

[31] Levi DM, Klein SA, Aitsebaomo AP. Vernier acuity, crowding and cortical magnification. Vision Res. 1985;25(7):963–77. . 4049746 10.1016/0042-6989(85)90207-x

[32] Tsai LT, Chen CC, Jang Y, Liao KM. The meridian effect on the cortical magnification factor for visual word form identification. J Vis. 2013;13(9):582.

[33] Miles WR. Ocular dominance in human adults. The Journal of General Psychology. 1930;3(3):412–30.

[34] Anstis SM. Letter: A chart demonstrating variations in acuity with retinal position. Vision Res. 1974;14(7):589–92. . 4419807 10.1016/0042-6989(74)90049-2

[35] Chan A, Lee P. Effect of display factors on Chinese reading times, comprehension scores and preferences. Behav Inf Technol. 2005;24(2):81–91.

[36] West BT, Welch KB, Gałecki AT. Linear Mixed Models: A Practical Guide Using Statistical Software. 2nd ed Boca Raton, FL: CRC Press; 2015.

[37] Portney LG, Watkins MP. Correlation Foundations of Clinical Research: Applications to Practice. Upper Saddle River, New Jersey: Prentice-Hall, Inc.; 2000 p. 491–508.

[38] Cheng G, Huang C, Deng H, Wang H. Diabetes as a risk factor for dementia and mild cognitive impairment: a meta-analysis of longitudinal studies. Intern Med J. 2012; 42(5):484–91. 10.1111/j.1445-5994.2012.02758.x . 22372522

[39] Biessels GJ, Staekenborg S, Brunner E, Brayne C, Scheltens P. Risk of dementia in diabetes mellitus: a systematic review. Lancet Neurol. 2006; 5(1):64–74. 10.1016/s1474-4422(05)70284-2 . 16361024

[40] Spry PG, Johnson CA, Mansberger SL, Cioffi GA. Psychophysical investigation of ganglion cell loss in early glaucoma. J Glaucoma. 2005; 14(1):11–9. . 15650598 10.1097/01.ijg.0000145813.46848.b8

[41] Chen KL, Hsieh CL, Sheu CF, Hu FC, Tseng MH. Reliability and validity of a Chinese version of the Pediatric Evaluation of Disability Inventory in children with cerebral palsy. J Rehabil Med. 2009; 41(4):273–8. 10.2340/16501977-0319 . 19247548

[42] Rizzo MR, Marfella R, Barbieri M, Boccardi V, Vestini F, Lettieri B, et al Relationships between daily acute glucose fluctuations and cognitive performance among aged type 2 diabetic patients. Diabetes Care. 2010; 33(10):2169–74. 10.2337/dc10-0389 . 20573753 PMC2945154

[43] Schreiber AK, Nones CF, Reis RC, Chichorro JG, Cunha JM. Diabetic neuropathic pain: Physiopathology and treatment. World J Diabetes. 2015; 6(3):432–44. 10.4239/wjd.v6.i3.432 . 25897354 PMC4398900

[44] Crassini B, Brown B, Bowman K. Age-related changes in contrast sensitivity in central and peripheral retina. Perception. 1988; 17(3):315–32. . 3067210 10.1068/p170315

[45] Scialfa CT, Cordazzo S, Bubric K, Lyon J. Aging and visual crowding. J Gerontol B Psychol Sci Soc Sci. 2013; 68(4):522–8. 10.1093/geronb/gbs086 . 23009956

[46] Billino J, Bremmer F, Gegenfurtner KR. Differential aging of motion processing mechanisms: evidence against general perceptual decline. Vision Res. 2008; 48(10):1254–61. 10.1016/j.visres.2008.02.014 . 18396307

[47] Felmingham KL, Baguley IJ, Green AM. Effects of diffuse axonal injury on speed of information processing following severe traumatic brain injury. Neuropsychology. 2004; 18(3):564–71. 10.1037/0894-4105.18.3.564 . 15291734

