# Supplementary material for: Detecting Visual Function Abnormality with a Contrast-Dependent Visual Test in Patients with Type 2 Diabetes
Source: PLoS One. 2016 Sep 9;11(9):e0162383. doi: 10.1371/journal.pone.0162383 (PMC5017771; doi:10.1371/journal.pone.0162383)
Supplement: S1 Appendix — (DOCX) [file pone.0162383.s001.docx]

**S1 Appendix.** Details of the calculation of the pooled ICCs from the three-level linear mixed-effects model

=============================================================

# Variable List:

"Male",

# Interaction:

"OV.x.Contrast_80",

"OV.x.Contrast_25",

"OV.x.Contrast_10",

"OV.x.Contrast_5",

# Multiple GAM:

"age.b48.703.s69.784",

"age.b69.784",

"age.s48.703",

# Simple GAM:

"age.b49.855.s70.814",

"age.b70.814",

"age.s49.855",

# Interaction:

"age.s48.703.x.Contrast_80",

"age.s48.703.x.Contrast_25",

"age.s48.703.x.Contrast_10",

"age.s48.703.x.Contrast_5",

"age.s48.703.rev.x.Contrast_80",

"age.s48.703.rev.x.Contrast_25",

"age.s48.703.rev.x.Contrast_10",

"age.s48.703.rev.x.Contrast_5",

"age.s48.703.x.OV.x.Contrast_80",

"age.s48.703.x.OV.x.Contrast_25",

"age.s48.703.x.OV.x.Contrast_10",

"age.s48.703.x.OV.x.Contrast_5",

"age.s48.703.rev.x.OV.x.Contrast_80",

"age.s48.703.rev.x.OV.x.Contrast_25",

"age.s48.703.rev.x.OV.x.Contrast_10",

"age.s48.703.rev.x.OV.x.Contrast_5"

=============================================================

# Three-Level Linear Mixed-Effects Model:

> summary(OV.std.GLMM.1)

Linear mixed-effects model fit by REML

Data: Longdata2

AIC BIC logLik

1502.982 1541.909 -743.4911

Random effects:

Formula: ~1 | ID_new

(Intercept)

StdDev: 0.9093767

Formula: ~1 | Contrast %in% ID_new

(Intercept) Residual

StdDev: 1.776053e-05 0.4105531

Fixed effects: OV.std ~ O + Contrast25 + Contrast10 + Contrast5

Value Std.Error DF t-value p-value

(Intercept) -0.003417820 0.08743215 475 -0.0390911 0.9688

O 0.006533199 0.02647079 475 0.2468079 0.8052

Contrast25 0.001301990 0.03721412 363 0.0349864 0.9721

Contrast10 -0.005582007 0.03721328 363 -0.1500004 0.8808

Contrast5 0.020273033 0.03762270 363 0.5388511 0.5903

Correlation:

(Intr) O Cntr25 Cntr10

O -0.151

Contrast25 -0.212 -0.001

Contrast10 -0.213 0.002 0.499

Contrast5 -0.213 0.017 0.494 0.493

Standardized Within-Group Residuals:

Min Q1 Med Q3 Max

-3.918375518 -0.474518973 -0.003292314 0.542474965 4.644379060

Number of Observations: 964

Number of Groups:

ID_new Contrast %in% ID_new

122 488

----------------------------------------------------------------------------------------------------

Calculation:

Pooled ICC = (0.9093767^2 + (1.776053e-05)^2) / (0.9093767^2 + (1.776053e-05)^2 + 0.4105531^2)

= 0.8306876
