# Supplementary figures and images for: Detecting Visual Function Abnormality with a Contrast-Dependent Visual Test in Patients with Type 2 Diabetes
Source: PLoS One. 2016 Sep 9;11(9):e0162383. doi: 10.1371/journal.pone.0162383 (PMC5017771; doi:10.1371/journal.pone.0162383)

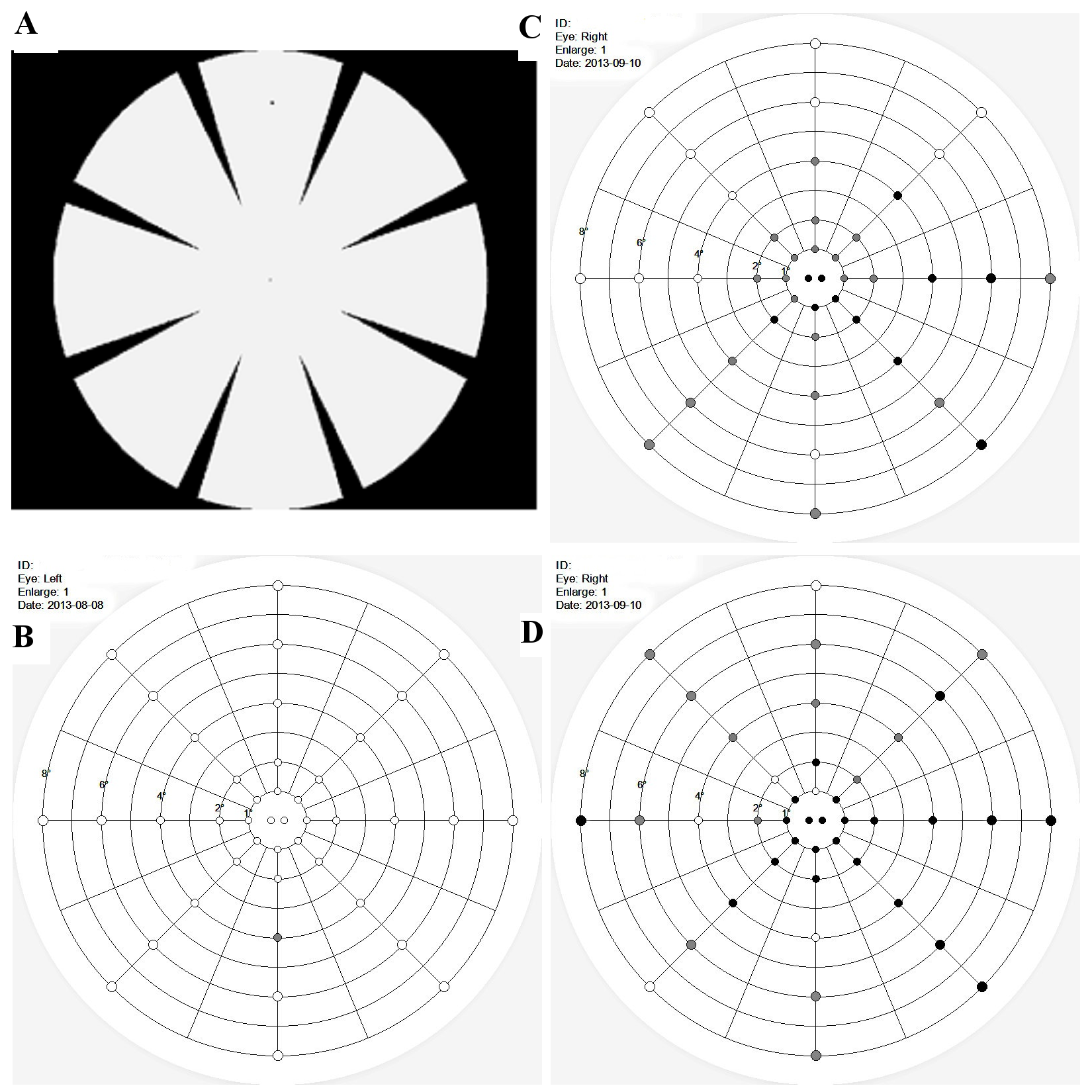

Supplement: S1 Fig — (A) Testing platform; (B) results of the MMFA in a representative healthy participant; (C) a type 2 diabetic patient with proliferative diabetic retinopathy tested at 25% contrast; and (D) the same patient tested at 10% contrast. The testing platform of the MMFA uses an eight-spoke wagon wheel to help stabilize the observer’s fixation. Visual stimuli are presented on the white wagon wheel (A). White dots represent correct identification (2 points). Grey dots represent incorrect responses (incorrect identification (1 point) or detection of a blurred image (0.5 point)). Black dots indicate that the participant did not detect anything at all (0 point) (B to D). In Fig 1(C), for example, there are 11 white dots (equal to 22 points), 20 grey dots (9 incorrect identifications (9 points) and 11 detecting blurred images (5.5 points)), and 11 black dots (0 point). Therefore, the total score of Fig 1(C) is 36.5 points. Compared with the control B) and between (C) and (D), this diabetic patient showed consistently poorer performance in the foveal, temporal, and lower fields. In addition, the more the contrast level decreased, the greater number of incorrect responses that occurred in the testing results. (TIF) [file pone.0162383.s002.tif]

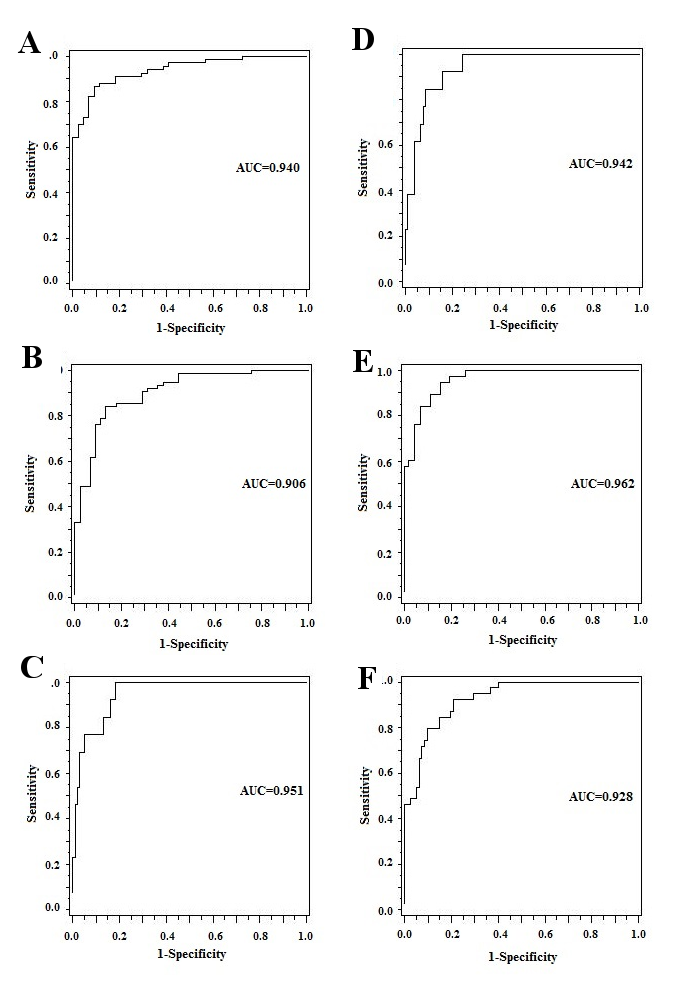

Supplement: S2 Fig — (TIF) [file pone.0162383.s003.tif]

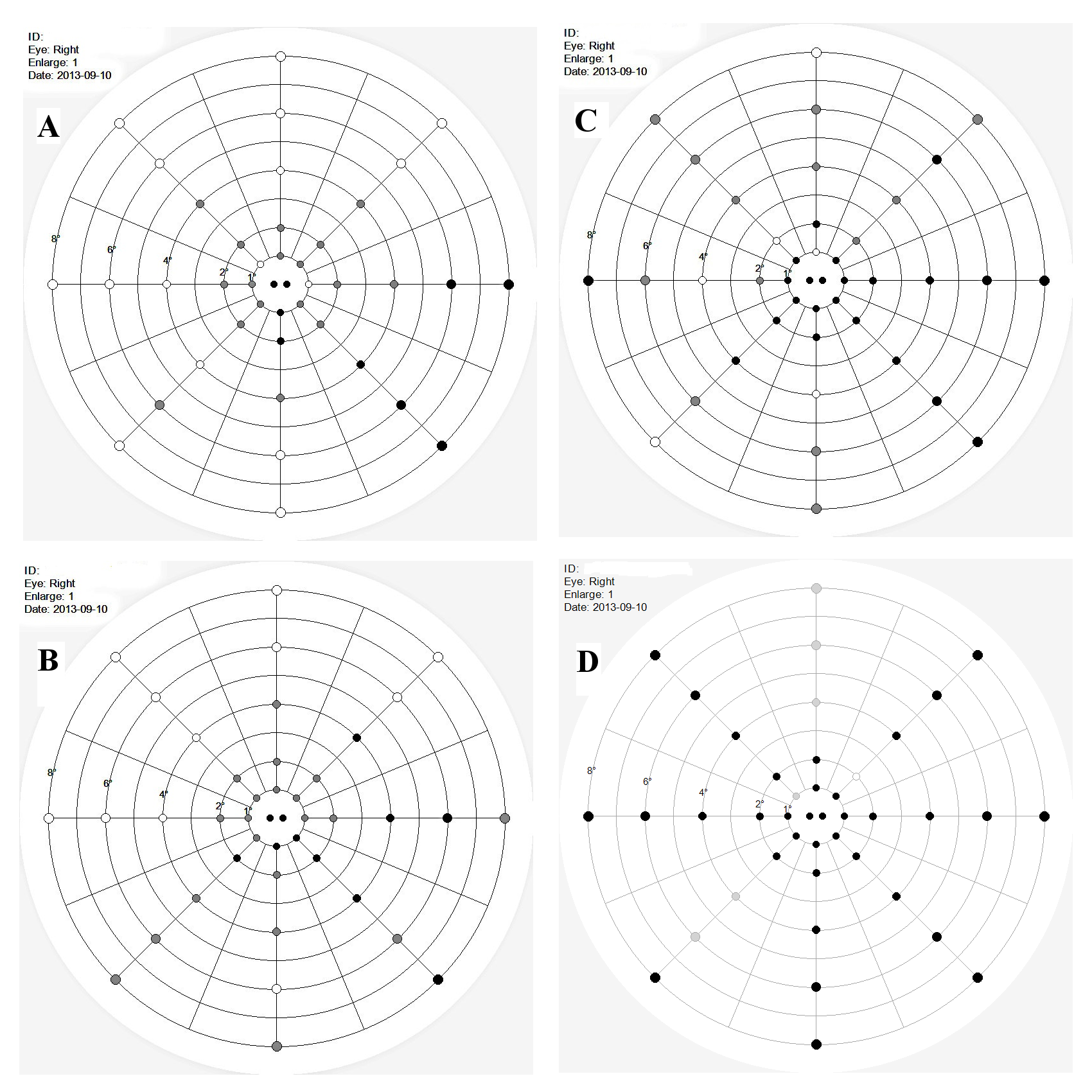

Supplement: S3 Fig — The contrast acuity values tested by the ETDRS test at 100%, 25%, 10%, and 5% contrast were 1.00, 0.75, 0.32, and 0.43 (in decimal notation), respectively. Although this patient had PDR and impaired performance in the MMFA, he still performed well on the ETDRS test. (TIF) [file pone.0162383.s004.tif]
