# Supplementary material for: Detecting Visual Function Abnormality with a Contrast-Dependent Visual Test in Patients with Type 2 Diabetes
Source: PLoS One. 2016 Sep 9;11(9):e0162383. doi: 10.1371/journal.pone.0162383 (PMC5017771; doi:10.1371/journal.pone.0162383)
Supplement: S1 Table — (PDF) [file pone.0162383.s005.pdf]

S1 Table. Chinese character, configuration, and stroke density used in the MMFA

Set	Configuration	Chinese Character	Stroke Density
I	Single	大 (da)	18.8
		太 (tai)	20.6
		天 (tian)	21.9
II	Left-right	仕 (shi)	24.0
		仟 (qian)	23.2
		任 (ren)	26.6
III	Top-down	古 (gu)	22.0
		占 (zhan)	20.5
IV	Surrounding	目 (mu)	24.2
		旦 (dan)	23.5
		貝 (bei)	26.9
V	Left-right	汙 (wu)	21.0
		江 (jiang)	20.3
		汪 (wang)	23.5

The stroke density was expressed as a pixel ratio of bit maps of the character images: the total pixels of the strokes to whole background image pixels. The character image size was 159 × 159 pixels. Similar pixel ratios presented similar difficulty in identifying these characters. The characters in each set had similar configurations and similar stroke densities. These characters were randomly selected on the MMFA testing plate by computer software.
