# Supplementary material for: Detecting Visual Function Abnormality with a Contrast-Dependent Visual Test in Patients with Type 2 Diabetes
Source: PLoS One. 2016 Sep 9;11(9):e0162383. doi: 10.1371/journal.pone.0162383 (PMC5017771; doi:10.1371/journal.pone.0162383)
Supplement: S2 Table — (PDF) [file pone.0162383.s006.pdf]

S2 Table. Parameters (ω_{T0} and E_2) used in the MMFA

ω_{T0}	Nasal	Temporal	Upper	Lower
80%	0.35	0.35	0.35	0.35
25%	0.48	0.48	0.48	0.48
10%	0.70	0.70	0.70	0.70
5%	0.78	0.78	0.78	0.78
E_2	Nasal	Temporal	Upper	Lower
80%	5.79	5.03	2.54	3.61
25%	5.84	5.28	3.13	4.14
10%	5.55	5.23	3.68	4.48
5%	5.63	5.79	4.88	5.03

ω_{T0} is the size threshold (degree of visual angle) in the center of the fovea, and E_2 is the cortical magnification factor. These variables would affect the minimum required size threshold to correctly discriminate the character. This table demonstrated the parameters (ω_{T0} and E_2) used in the MMFA to adjust the character size at different eccentricities, meridians, and contrast levels.
