# Supplementary material for: Detecting Visual Function Abnormality with a Contrast-Dependent Visual Test in Patients with Type 2 Diabetes
Source: PLoS One. 2016 Sep 9;11(9):e0162383. doi: 10.1371/journal.pone.0162383 (PMC5017771; doi:10.1371/journal.pone.0162383)
Supplement: S3 Table — (PDF) [file pone.0162383.s007.pdf]

S3 Table. Comparing the results of multiple linear regression analysis of the ETDRS and MMFA scores at 80%, 25%, 10%, and 5% contrast levels for all participants.

Group	100% ETDRS			25% ETDRS			10% ETDRS			5% ETDRS		
	β	SE	*p* value	*β*	SE	*p* value	*β*	SE	*p* value	*β*	SE	*p* value
0												
1	0.108	0.043	0.014*	0.072	0.049	0.142	0.095	0.051	0.067	0.107	0.053	0.044*
2	0.151	0.052	0.005*	0.144	0.059	0.017*	0.225	0.063	0.001*	0.242	0.065	0.000*
3	0.110	0.099	0.270	0.111	0.112	0.321	0.090	0.118	0.450	0.095	0.122	0.436
4	0.332	0.075	<0.001*	0.291	0.084	0.001*	0.303	0.089	0.001*	0.377	0.091	<0.001*
5	0.688	0.085	<0.001*	0.736	0.096	<0.001*	0.900	0.101	<0.001*	0.774	0.110	<0.001*

Group	80% MMFA			25% MMFA			10% MMFA			5% MMFA		
	β	SE	*p* value	*β*	SE	*p* value	*β*	SE	*p* value	*β*	SE	*p* value
0												
1	-7.44	3.75	0.050	-1.73	4.04	0.670	-2.70	4.49	0.540	-12.67	5.73	0.029*
2	-14.33	4.60	0.002*	-10.96	4.96	0.029*	-13.10	5.38	0.017*	-14.67	6.68	0.030*
3	-21.89	8.64	0.013*	-23.62	9.32	0.013*	-32.90	10.12	<0.001*	-37.86	12.27	0.003*
4	-25.03	6.49	<0.001*	-17.26	7.00	0.015*	-30.58	7.60	<0.001*	-30.47	9.23	0.001*
5	-47.71	7.42	<0.001*	-52.36	8.00	<0.001*	-57.95	9.40	<0.001*	-41.19	10.61	<0.001*

Group 0 = non-DM control; Group 1= no-DR; Group 2 = NPDR; Group 3 = PDR; Group 4 = NPDR & CSME; Group 5 = PDR & CSME. Data from the controls (Group 0) were used as the baseline for comparison with that of the diabetic patients (Groups 1 to 5) with different retinal conditions. * indicates significant p values (i.e., $p < 0.05$).
